# Role of Dach1 revealed using a novel inner ear-specific Dach1-knockdown mouse model

**DOI:** 10.1242/bio.043612

**Published:** 2019-08-12

**Authors:** Toru Miwa, Ryosei Minoda, Yoshihide Ishikawa, Tomohito Kajii, Yorihisa Orita, Takahiro Ohyama

**Affiliations:** 1 Department of Otolaryngology and Head and Neck Surgery, Graduate of School of Medicine, Kumamoto University, Kumamoto 8608556, Japan; 2 Otolaryngology-Head and Neck Surgery, JCHO Kumamoto General Hospital, Kumamoto 8668660, Japan; 3 USC-Tina and Rick Caruso Department of Otolaryngology-Head & Neck Surgery, Zilkha Neurogenetic Institute, USC Keck School of Medicine, University of Southern California, Los Angeles, CA 90089, USA

**Keywords:** *Dach1*, Knockdown mouse, Stria vascularis, Intermediate cell, Epithelial-mesenchymal transition

## Abstract

The *Dach1* gene is expressed in the inner ear of normal mouse embryos in the area that differentiates into the cochlear stria vascularis (SV). We hypothesised that *Dach1* downregulation in the inner ear would lead to SV dysplasia. However, because *Dach1* knockout is embryonic lethal in mice, the role of *Dach1* in the inner ear is unclear. Here, we established inner ear-specific *Dach1*-knockdown mice and showed that *Dach1* downregulation resulted in hearing loss, reduced endocochlear potential and secondary outer hair cell loss. There were no abnormalities in marginal cells and basal cells in the SV or spiral ligament in inner ear-specific *Dach1*-knockdown mature mice. However, intermediate cell dysplasia and thinning of the SV were observed. Moreover, dynamic changes in the expression of key genes related to the epithelial-mesenchymal transition were observed in the lateral wall of the cochlear epithelium, which differentiated into the SV in inner ear-specific *Dach1*-knockdown mice at embryonic stages. In summary, suppression of *Dach1* expression in the inner ear caused the epithelial-mesenchymal transition in the lateral wall of cochlear epithelium, resulting in loss of intermediate cells in the SV and SV dysplasia.

This article has an associated First Person interview with the first author of the paper.

## Abstract (precis)

**Summary:** Inner ear-specific downregulation of Dach1 reveals that the epithelial–mesenchymal transition is crucial for the generation of the stria vascularis.

## INTRODUCTION

**Fig. 1.**
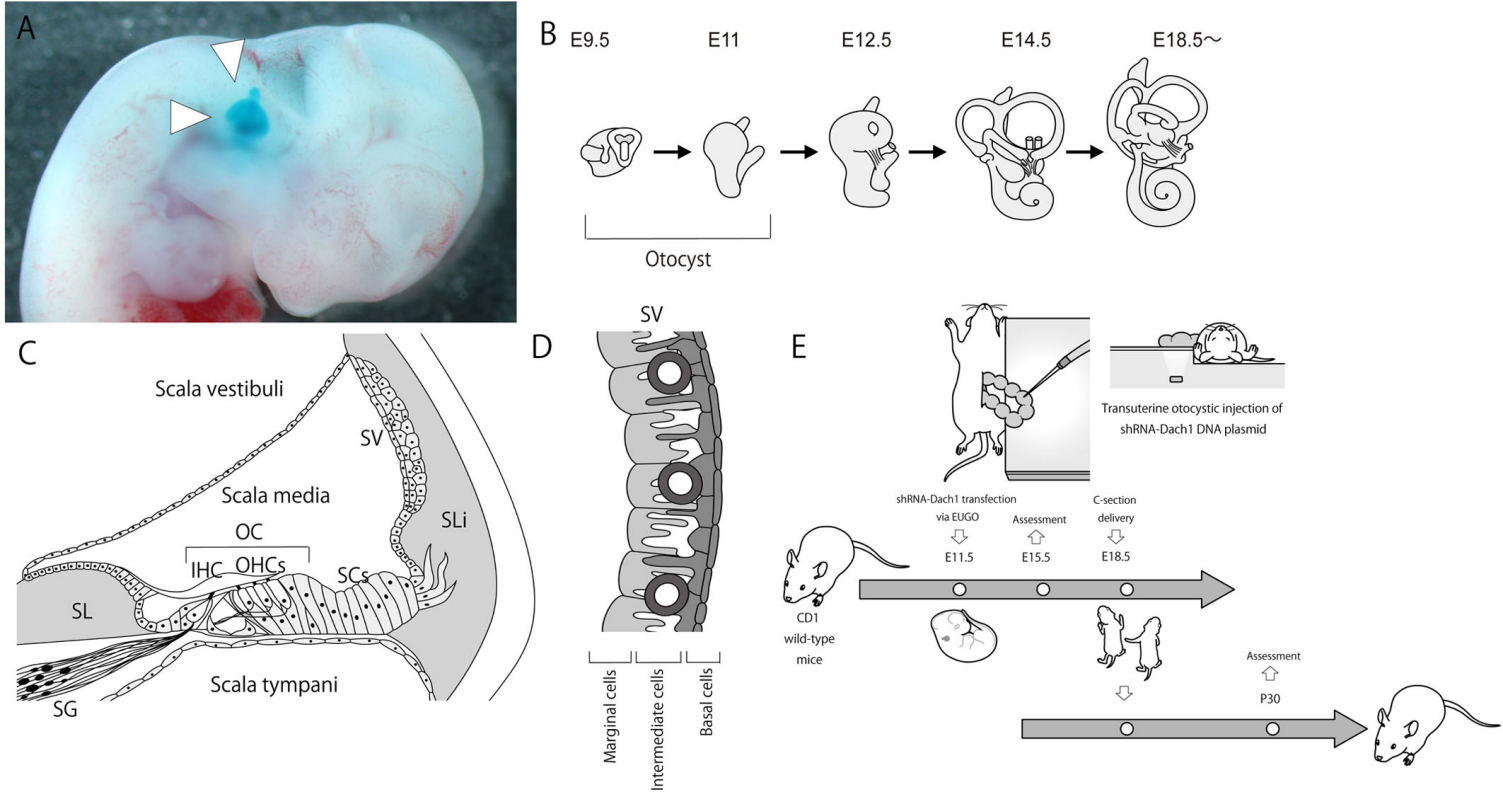
**Electroporation-mediated transuterine gene transfer into otocysts (EUGO) in mice at E11.5.** (A) The arrowhead indicates an E11.5 embryo otocyst. Fast Green dye was microinjected into the otocysts, and the uterine wall was then removed. (B) Development of cochlea. (C) A cross-sectional image of a typical adult cochlea. The adult mammalian cochlea is divided into three compartments: the scala vestibuli, scala tympani and scala media. Depicted here is a cross-section of the scala media, which contains the organ of Corti (OC). The OC contains three types of cell populations: inner hair cells (IHCs), outer hair cells (OHCs) and supporting cells (SCs). The two types of auditory hair cells play critical roles in hearing as mechanoelectrical transducers. The auditory hair cells are overlaid by the tectorial membrane (TM). The SV, in the lateral wall of the scala media, is responsible for the secretion of K+ into the endolymph and for endocochlear potential production. SL, spiral limbus; SLi, spiral ligament; SG, spiral ganglion. (D) Scheme of the cellular structure of the SV. Cell types are as indicated in the figure. SV integrity relies on a three-layered tissue architecture of marginal, intermediate and basal cells that enclose a dense capillary network. The formation of long cell processes by all three cell types and a high degree of interdigitation characterises strial architecture. (E) EUGO was performed in mice at E11.5. Embryos were delivered via C-section at E18.5, and the pups that underwent gene transfer at E11.5 were passed to surrogate dams.

The molecular mechanisms required for the development of inner ear tissues to enable normal hearing sensory function are unclear (Heller, 2013). The Dachshund family transcription factor 1 (*Dach1*) gene is the mammalian homolog of the *Drosophila* dachshund gene (*dac*). Notably, in *Drosophila*, loss of or damage to the *dac* gene causes hypoplasia of the eyes and limbs (Davis et al., 2001), and the *dac* gene works as a differentiation factor in various tissues (Okamoto et al., 2012). In the inner ear of normal mouse embryos, *Dach1* is known to be expressed in the dorsal otocyst, a sac-like epithelial structure (Fig. 1A,B), at embryonic day (E)12 and the lateral wall of the cochlear duct epithelium, which is the region that differentiates into the stria vascularis (SV) at E15 (Ayres et al., 2001). The SV is comprised of three layers: the marginal, intermediate and basal cell layers that enclose a dense capillary network (Fig. 1C,D). Previous reports have shown that deletion of genes that control the formation of marginal, intermediate and basal cells causes reduction of endocochlear potential (EP) and presentation of hearing loss (Delpire et al., 1999; Erichsen et al., 1996; Gow et al., 2004; Kitajiri et al., 2004; Minowa et al., 1999; Phippard et al., 1999; Shibata et al., 2016; Trowe et al., 2008; Vetter et al., 1996). EP is required for normal hearing sensation and is generated via potassium-ion exchange conducted by epithelial-mesenchymal cell networks in the SV and the spiral ligament (Kikuchi and Hilding, 1966). Although the *Dach1* gene is thought to play a crucial role in the formation of SV, which is formed postnatally, few studies have investigated this role, as *Dach1* deletion causes embryonic lethality in mice (Ayres et al., 2001). While studies knocking out embryonically expressed genes in mice are useful for clarifying the functions of many genes, when gene knockout causes embryonic lethality, it is necessary to utilise organ-specific expression models [conditional knockout (cKO)], which can be time-consuming and expensive to establish. Our group has recently achieved suppression of target gene expression through transfer of short hairpin RNA (shRNA) plasmids into otocysts at E11.5. In our previous study, we showed that vectors transfected in this manner continued to be expressed for about 1 month (Minoda et al., 2015; Miwa et al., 2013). Thus, our technique has established a method for the production of an inner ear-specific gene-expression suppression model in mature mice (Miwa et al., 2013) (Fig. 1E). Except for the technical difficulty of gene transfer into otocysts, our method is much more convenient, quick and inexpensive than the traditional cKO method.

Accordingly, we hypothesised that Dach1 downregulation in the inner ear would lead to SV dysplasia, resulting in an EP drop and congenital hearing loss. Therefore, in this study, we aimed to determine the role of *Dach1* in the inner ear of mice by establishing an inner ear-specific *Dach1*-knockdown (KD) mouse and evaluating the effects of *Dach1* on hearing in these mice.

## RESULTS

### The shRNA-Dach1 plasmid was expressed in the treated developing cochlear duct

**Fig. 2.**
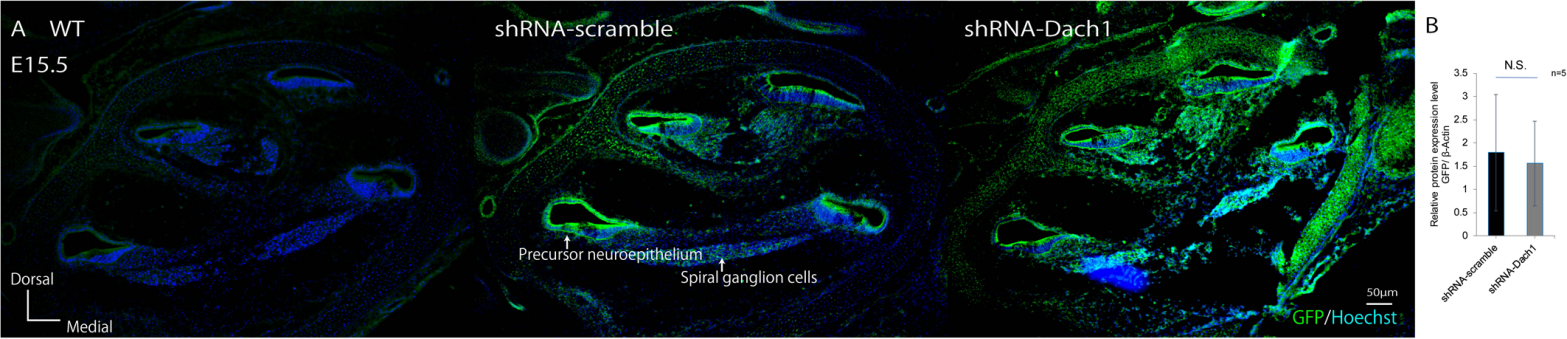
**The shRNA-Dach1 plasmid was expressed in the treated developing cochlear duct.** (A) After injecting the GFP-containing shRNA-Dach1 plasmid and the GFP-containing shRNA-scramble plasmid into the inner ear of a wild-type (WT) mouse embryo, GFP signals were observed in the precursor neuroepithelium, medial wall, lateral wall and the spiral ganglion cells in the inner ear at E15.5. (B) No difference was observed in the expression of GFP between groups transfected with shRNA-scramble and shRNA-Dach1 (*P*=0.44). Scale bar: 50 μm. N.S., not significant.

After injecting the green fluorescent protein (GFP)-containing shRNA-Dach1 plasmid, which encoded siRNAs against the *Dach1* gene, and the GFP-containing shRNA-scramble plasmid, which carried randomised sequences and was used as a control vector, into the inner ear of a wild-type mouse embryo, electroporation-mediated transuterine gene transfection into otocysts (EUGO) was performed at E11.5. The treated embryos were removed and used for histological assessments at E15.5. Frozen-cryosection and immunohistological analysis revealed GFP signals in the precursor neuroepithelium, medial wall, and lateral wall of cochlear duct epithelial and spiral ganglion cells, whereas no GFP signal was observed in wild-type untreated mice at E15.5 (Fig. 2A). There were no differences in GFP signal levels between the shRNA-Dach1 plasmid group and control plasmid group according to western blot analysis (Fig. 2B).

### The Dach1 signal was lost in the treated cochleae

**Fig. 3.**
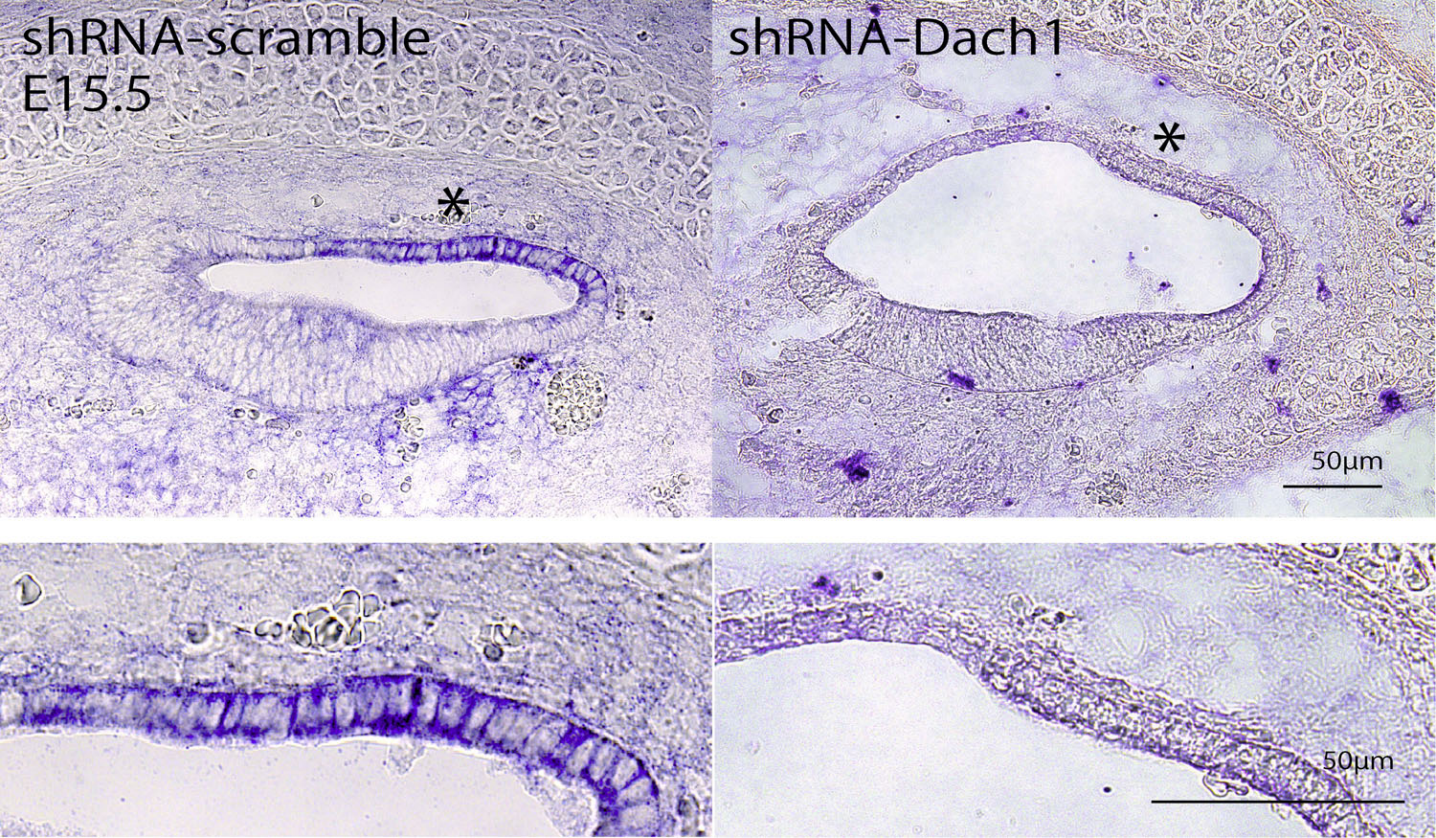
**The *Dach1* signal was lost in the treated cochleae.** After transferring the shRNA-Dach1 plasmid, *Dach1* mRNA expression was lost in the lateral wall of the cochlear duct at E15.5 (asterisks), and the cochlear duct was observed to balloon slightly. The images shown in the lower panels are higher magnification images of the lateral wall. All images were taken in the middle turns of the cochlear duct.

After gene transfection with the shRNA-Dach1 plasmid at E11.5, the treated embryos were removed and used for histological assessments at E15.5. After cryosectioning, we utilised *in situ* hybridisation to examine *Dach1* expression inside the embryonic inner ear at E15.5. We observed ballooning of the cochlear duct and loss of *Dach1* mRNA expression in the lateral wall in the inner ear-specific Dach1-KD mice compared with that in the control mice at all cochlear turns (Fig. 3, middle turn images are shown).

### Intermediate cells were absent from the SV of the cochlea of inner ear-specific Dach1-KD mice

**Fig. 4.**
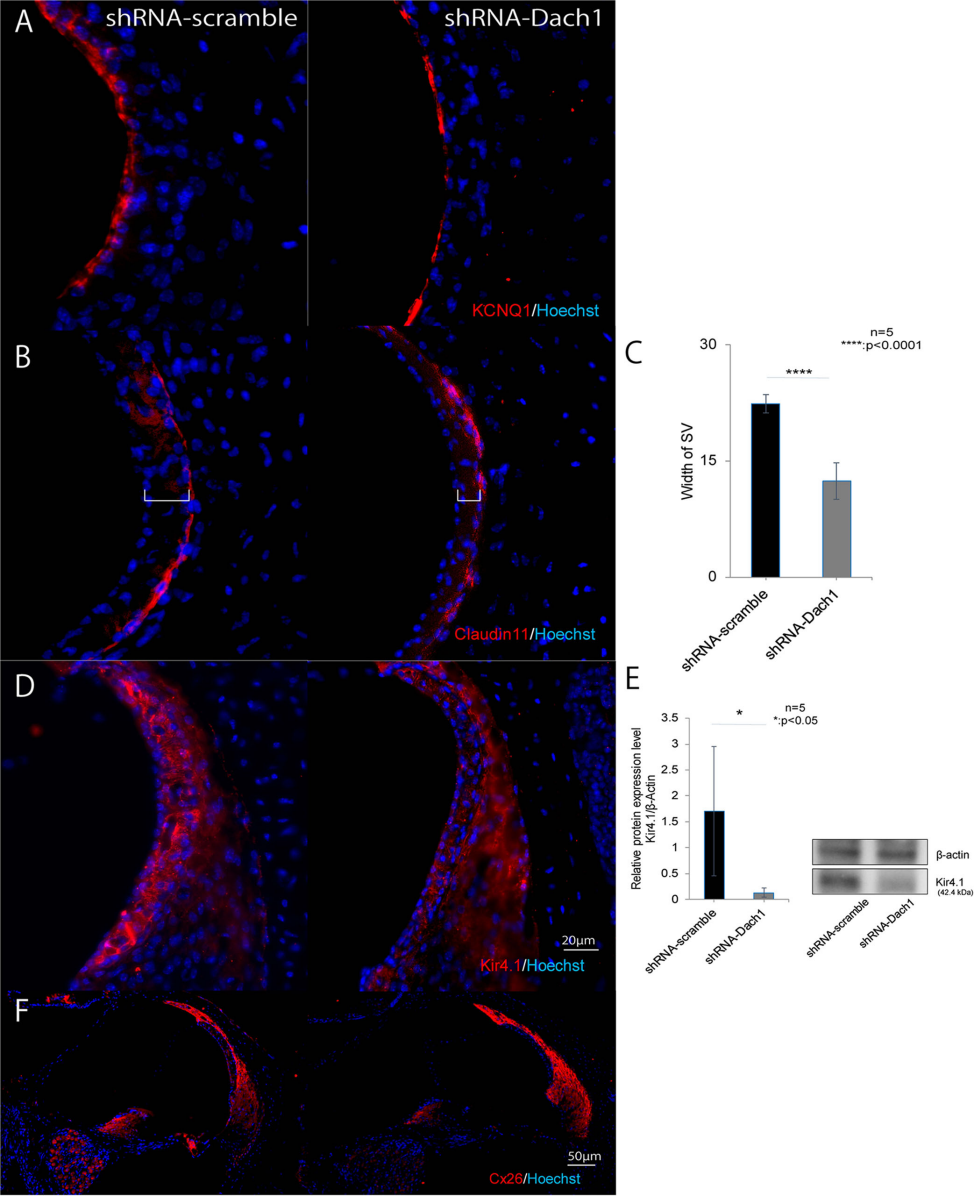
**Intermediate cells were absent from the SV of the cochlea of inner ear-specific Dach1-KD mice.** (A,B) Expression of the potassium channel KCNQ1, a marginal cell marker (A), and Claudin11, a basal cell marker (B), in mature inner ear-specific Dach1-KD mice and control mice. No difference was observed in the expression of KNCQ1 and Claudin11 between control mice and KD mice. (C) Thickness of the SV of KD mice was significantly decreased compared to control mice (****P<0.0001). (D,E) Expression of Kir4.1, a marker of intermediate cells in KD mice was significantly decreased, as confirmed by western blotting (*P=0.01). (F) No difference was observed in the expression of Connexin26, a marker of the spiral ligament between KD mice and control mice. Square brackets show the thickness of SV.

After gene transfection with the shRNA-Dach1 plasmid at E11.5, the treated embryos were delivered via Caesarean section and were cared for by surrogate mothers until postnatal day (P)30. The inner ears were dissected from adult temporal bones, and cryosectioning and immunohistological analyses were performed. There were no differences in the expression of the potassium channel KCNQ1 (Yang et al., 2013), a marginal cell marker (Fig. 4A), or Claudin11, a basal cell marker (Kitajiri et al., 2004) (Fig. 4B), in mature inner ear-specific Dach1-KD mice compared with that in the control group. However, the SV of KD mice was noticeably thinner (Fig. 4C, *P*<0.0001). The expression of Kir4.1, a marker of intermediate cells (Marcus et al., 2002), was lost (Fig. 4D), as confirmed by western blotting (Fig. 4E, *P*=0.01). No differences in expression of Cx26, a marker of the spiral ligament (Forge et al., 2003), were observed between KD mice and control mice (Fig. 4F).

### The epithelial-to-mesenchymal transition (EMT) was induced in the lateral wall of the treated developing cochlear duct

**Fig. 5.**
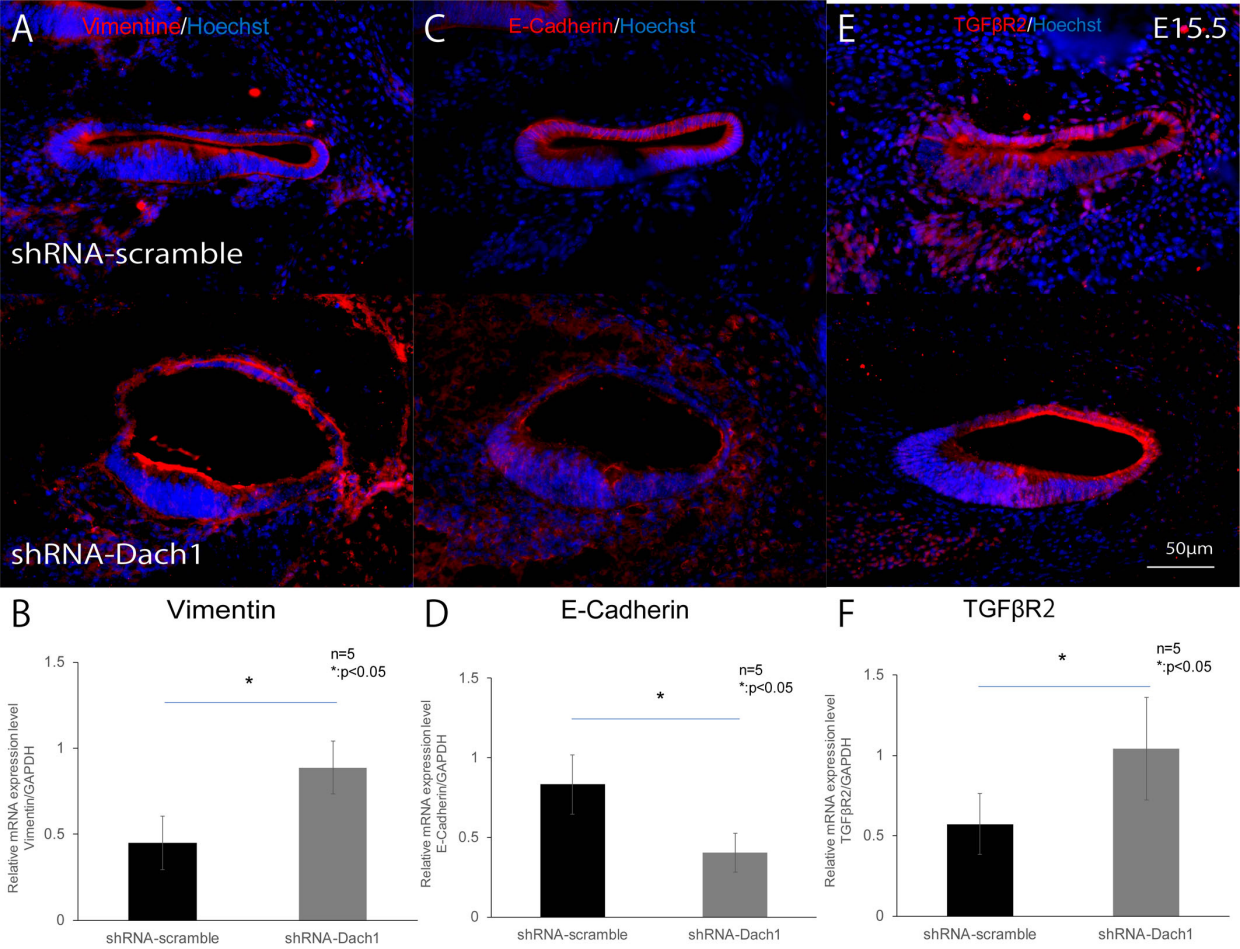
**The EMT in the lateral wall of the treated developing cochlear duct.** (A–F) Protein and mRNA expression in the lateral wall of the cochlear epithelium, utilising immunohistochemistry and qRT-PCR analyses in inner ear-specific Dach1-KD mouse embryos and control embryos at E15.5. (A,B) Vimentin (*P=0.03) (C,D) E-cadherin (*P=0.04). (E,F) TGFβR2 (*P=0.04).

After gene transfection with the shRNA-Dach1 plasmid at E11.5, the treated embryos were removed and used for histological assessments at E15.5. After cryosectioning in the lateral wall of the cochlear epithelium of inner ear-specific Dach1-KD mouse embryos, immunohistological assessment and quantitative reverse transcription polymerase chain reaction (qRT-PCR) analyses following laser microdissection (LMD) and extraction of lateral wall cells, a significant increase in vimentin expression at E15.5 (Fig. 5A,B, *P*=0.03) and a significant decrease in E-cadherin expression at E15.5 (Fig. 5C,D, *P*=0.04) was revealed. In addition, we observed a significant increase in transforming growth factor β receptor 2 (TGFβR2) expression at E15.5 (Fig. 5E,F, *P*=0.04).

### Loss of Dach1 signalling in the cochlea caused reduced EP and hearing loss

**Fig. 6.**
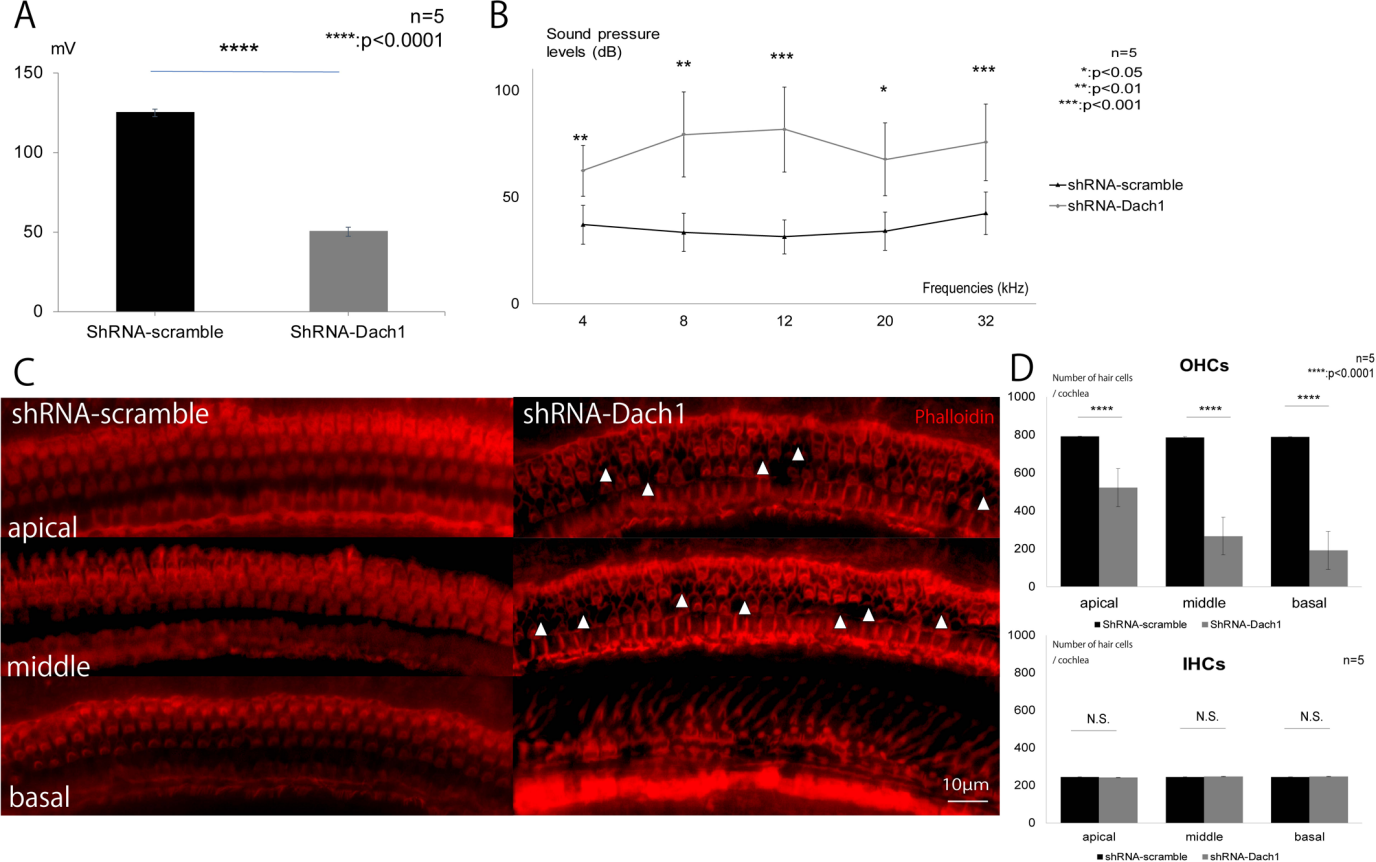
**Loss of Dach1 signalling in the cochlea caused decreased EP and deafness.** (A) EP in mature inner ear-specific Dach1-KD mice and control mice (****P<0.0001). (B) Hearing thresholds in mature inner ear-specific Dach1-KD mice and control mice (4 kHz, **P=0.003; 8 kHz, **P=0.004; 12 kHz, ***P=0.0009; 20 kHz, *P=0.01; 32 kHz, ***P=0.0005). (C,D) Auditory outer hair cells, but not inner hair cells, were malformed and degenerated in the cochleae of adult inner ear-specific Dach1-KD mice in the absence of Dach1 signalling. White arrowheads show the loss of hair cells. ****P<0.0001; N.S., not significant.

In mature inner ear-specific Dach1-KD mice at P30, EP recordings and auditory brainstem response recordings were performed under general anaesthesia. We observed a significant decrease in EP compared with that in the control mice (Fig. 6A, *P*=0.0005). Moreover, significant increases in hearing threshold values were observed at all frequencies compared with those in control mice (Fig. 6B: 4 kHz, *P*=0.003; 8 kHz, *P*=0.004; 12 kHz, *P*=0.0009; 20 kHz, *P*=0.01; 32 kHz, *P*=0.0005).

### Auditory hair cells were malformed and degenerated in the cochleae of mature inner ear-specific Dach1-KD mice in the absence of Dach1 signalling

In mature inner ear-specific Dach1-KD mice at P30, the bony capsule and lateral wall of the cochlea were removed and the surface morphology of the cochlea was assessed by immunostaining of phalloidin and counting of hair cells. We observed a significant reduction in the number of outer hair cells at all turns (Fig. 6C,D: apical, *P*=0.0003; middle, *P*=0.0002; basal, *P*=0.0009), but not of inner hair cells compared with control mice (Fig. 6C,D: apical, *P*=0.14; middle, *P*=0.34; basal, *P*=0.07).

## DISCUSSION

Our investigation of inner ear-specific Dach1-KD mature mice, which were generated by an electroporation method (Brigande et al., 2009; Gubbels et al., 2008) to transfect a gene vector containing an RNA interference cassette at E11.5, before the normal *Dach1* gene is expressed in the inner ear of the embryo, revealed that suppression of Dach1 expression caused clear hearing loss, reduced EP, and resulted in loss of outer hair cells compared with the control group. In addition, histological analysis revealed no abnormalities in the marginal cells (KCNQ1) (Yang et al., 2013), basal cells (Claudin11) (Kitajiri et al., 2004) or spiral ligament (Cx26) (Forge et al., 2003) of mature inner ear-specific Dach1-KD mice; however, we observed dysplasia of intermediate cells (Kir4.1) (Marcus et al., 2002) and thinning of the SV. Intermediate cells are derived from neural crest cells and are epithelial cells that divide the endolymph in the cochlear duct (Forge and Wright, 2002; Raphael and Altschuler, 2003). Dysplasia of the intermediate cell layer causes hearing loss (Shibata et al., 2016; Trowe et al., 2008). Loss of genes that are needed to form the spiral ligament also causes loss of EP (Delprat et al., 2005; Forge et al., 2003; Kikuchi et al., 2000; Teubner et al., 2003). From these findings, we concluded that reduction of EP due to dysplasia of the SV and secondary loss of outer hair cells caused the observed hearing loss (Marcus et al., 2002; Trowe et al., 2008).

Next, in our examination of inner ear-specific Dach1-KD mouse embryos, we observed an increase in the expression of vimentin, a marker of mesenchymal cells, and decreases in the expression of E-cadherin, a marker of epithelial cells in the lateral wall of cochlear epithelium, which differentiated to SV at embryonic stage. In addition, we observed increased TGFβR2, which is involved in TFGβ signalling, in the lateral wall of the cochlear epithelium at embryonic stages. This expression pattern is indicative of the EMT, which, in addition to being important in embryonic development, plays a crucial role in the invasion and metastasis of cancer cells (Thiery and Sleeman, 2006). In progressive breast cancer tissues, the Dach1 signal is lost, and decreases in E-cadherin expression, loss of intercellular adhesion and loss of epithelial morphology have been observed accompanying increases in metastasis and infiltration (Thiery and Sleeman, 2006; Vanderburg and Hay, 1996). In other words, during the EMT, the levels of the mesenchymal cell-specific intermediate filament protein vimentin increased, whereas the levels of the intercellular adhesion protein E-cadherin decreased, thereby facilitating the conversion of epithelial cells to mesenchymal cells (Yang et al., 2004); this results in the loss of intercellular adhesion and epithelial morphology, such as is observed in progressive breast cancer. Furthermore, the TGFβ signalling pathway, which has major roles in the EMT (Kalluri and Weinberg, 2009), is suppressed by Dach1 via binding to Smad4 in breast cancer tissue (Wang, 2015; Wu et al., 2003), thereby controlling the EMT. Finally, intermediate cells normally arise from neural crest cells that differentiate into melanocytes and are incorporated into the SV (Shibata et al., 2016). However, previous studies have reported that E-cadherin disruption causes dysplasia of intermediate cells in the SV (Trowe et al., 2011). Based on these findings, we concluded that in our inner ear-specific Dach1-KD mouse embryos, suppression of Dach1 in the cochlear epithelial lateral wall cells caused the EMT, resulting in lack of formation of intermediate cells and causing SV dysplasia. In summary, our findings provide critical insights into the non-autonomous bystander effect and roles of Dach1 in the inner ear. However, this study is limited by the fact that correlations with other dysplasia-causing genes in intermediate cells in the SV and the detailed mechanisms of EMT are not fully examined. In addition, we used an shRNA-Dach1 plasmid to generate KD mice, and the shRNA system can induce off-target effects, although we checked for similar sequences in the genome using BLAST in an attempt to limit such effects. Accordingly, further studies are needed to investigate the genesis of intermediate cells in the SV using inner-ear specific Dach1-knockout mice.

## MATERIALS AND METHODS

### Ethics statement

All animal experiments were approved by the Committee on the Use and Care of Animals at Kumamoto University, Japan, and were performed in accordance with accepted veterinary standards (Number: H28-053).

### Animals

Normal 6–8-week-old CD-1-timed pregnant *Mus musculus* were purchased from Kyudo (Kumamoto, Japan). Animals were housed in a temperature-controlled room maintained at about 25°C with a humidity of about 50%. A standard commercial pellet diet and water were given to all animals *ad libitum*. Throughout the study, the numbers of ears and mice analysed were 60 and 30, respectively.

### Plasmid vectors

All plasmids were purchased from GeneCopoeia (Rockville, MD, USA). shRNA-Dach1 encoded siRNAs against the *Dach1* gene. The siRNA target sequences for the mouse *Dach1* gene were: aagtggcttcctttacggt, accttagcaccattgcaaa, gtccatgaaccagatgctt and cagtggtggttcttgggat. We used BLAST to check the similarity of the sequences with those in the international nucleotide database to confirm the specificities of the four Dach1-target shRNAs. A scrambled plasmid that carried randomised sequences was used as a control vector for shRNA-Dach1. To generate the shRNA-Dach1 scrambled plasmid, the Gateway vector psi-U6 (containing the U6 promoter and an eGFP tag), and Gateway-compatible ORFEXPRESS shuttle clones corresponding to each gene were subcloned. These expression plasmids were prepared with a Qiagen Endofree Plasmid Maxi Kit (Qiagen, Valencia, CA, USA) following the manufacturer's instructions. The plasmids were passed through a 0.22-μm filter, precipitated with ethanol, resuspended in sterile phosphate-buffered saline at a concentration of 3 μg/μl and stored at −20°C.

### Gene transfer to the embryonic inner ear

Gene transfer to the embryonic inner ear was performed as described in previous reports (Minoda et al., 2015; Miwa et al., 2013). At 11.5 days post-coitum, pregnant mice were deeply anaesthetised through intraperitoneal administration of 4 mg/kg xylazine (Bayer, Shawnee Mission, KS, USA) and 120 mg/kg ketamine-HCl (Daiichisankyo, Tokyo, Japan) in 0.9% NaCl. The uterus was exposed by a low-midline laparotomy, placed on a transparent surgical stage and illuminated from below with a fibre-optic beam to illuminate the rostral and caudal branches of the primary head vein, between which the otocyst was located. Plasmid vectors containing Fast Green dye (Sigma-Aldrich) were microinjected by oral pressure into the lumens of the unilateral otocysts of one or two embryos per dam using heat-pulled glass micropipettes (Fig. 1E). The plasmid-filled otocysts were then gently pinched on the right and left sides of the embryonic head using a 5-mm field from tweezer-style electrode paddles. Subsequently, the plasmid-filled otocysts were electroporated with 2×25 V biphasic poring pulses, 10 ms-long, at 950 ms intervals with decay rates from each antecedent pulse at 40%. To transfer plasmid into the cytoplasm, 2×50 ms-long biphasic transfer pulses at 15 V, pulse interval 950 ms and decay rate 40% (NEPA21 Electroporator; Nepa Gene, Chiba, Japan) were used. The abdominal skin was closed with Nesco 3-0 nylon sutures (Alfresa Pharma, Osaka, Japan).

### Experimental design

After gene transfection at E11.5, Caesarean sections were performed on the dams at E18.5. The treated embryos were removed, and surrogate mothers cared for the pups until postnatal evaluation at P30. These mice were defined as ‘mature inner ear-specific Dach1-KD mice’ and were assessed by auditory functional and morphological analyses of the cochleae. The other treated embryos were removed and used for histological assessments at E15.5 (Fig. 1E). The day of the Caesarean section was designated as P0. Noon was considered E0.5.

### *In situ* hybridisation analysis

Embryos were dissected and fixed in 4% paraformaldehyde (PFA) in phosphate-buffered saline (PBS) for 12 h at 4°C. For cryostat sectioning, the cochleae were embedded in OCT medium (Sakura Finetek Japan, Tokyo, Japan) and were serially sectioned to a thickness of 14 μm. *In situ* hybridisation analysis was performed following a standard procedure with digoxigenin-labelled antisense riboprobes (Moorman et al., 2001). Details of the probes used in this analysis available upon request. Sections were photographed using a BZ-9000 microscope (Keyence, Osaka, Japan).

### Immunohistochemistry

Embryos were dissected and fixed as described above. P30 mice were fixed by cardiac perfusion with 4% PFA in PBS, and the inner ears were dissected from adult temporal bones and decalcified in 0.5 M EDTA/PBS. For cryostat sectioning, the cochleae were serially sectioned to a thickness of 12 μm. For detection of antigens, the following primary antibodies and dilutions were used: anti-KCNQ1 (1:100; cat. no. SAB2501224; Sigma-Aldrich), anti-Claudin11 (1:100; cat. no. ab53041; Abcam), anti-Kir4.1 (1:200; cat. no. APC-035; Alomone Labs, Jerusalem, Israel), anti-Connexin26 (1:200; cat. no. 710500; Life Technologies), anti-Vimentin (1:50; cat. no. sc-7558; Santa Cruz Biotechnology), anti-E-cadherin (1:200; cat. no. 205604; Labome; Princeton, NJ, USA) and anti-TGFβR2 (1:100; cat. no. PA5-35076; Life Technologies). Fluorophore-coupled secondary antibodies were used at a dilution of 1:500. Labelling with the primary antibody was performed at 4°C overnight after blocking in 10% goat serum or 10% donkey serum for 10 min in PBS. Labelling with the secondary antibody was performed at room temperature for 1 h. Hoechst 33258 dye (Molecular Probes) was applied for 30 s for nuclear staining. The samples were examined under a BZ-9000 fluorescence microscope (Keyence). To study the surface morphology of the cochleae, P30 mice were fixed by cardiac perfusion with PFA/PBS under deep anaesthesia, and the bony capsule and lateral wall of the cochlea were removed. Texas Red-X phalloidin (1:100; Molecular Probes) was applied for 30 min, and the surface morphology of the cochleae was examined under a BZ-9000 fluorescence microscope.

### Hair cell counts

Following immunostaining with Texas Red-X phalloidin, five randomly selected surface images of the organ of Corti in each turn of the cochlea were captured at 40× magnification for each group. The numbers of inner hair cells and outer hair cells in a 140-μm basal segment of the basilar membrane were calculated for each group. Only the hair cells with an intact stereociliary bundle and a cuticular plate were counted.

### Auditory thresholds

We assessed the hearing thresholds at P30 by evaluating the auditory brainstem response (System 3; Tucker-Davis Technologies, Alachua, FL, USA). The animals were anaesthetised as described above. Electrodes were placed beneath the pinna of the test ear and at the vertex just below the surface of the skin. The ground electrode was placed under the contralateral ear. Auditory thresholds were measured at 4, 8, 12, 20 and 32 kHz by determining the auditory brainstem response (15 ms duration, 1 ms rise/fall time and tone burst). For each recording, an average of 512 sweeps was calculated. The stimulus levels near the threshold were varied in 10-dB steps, and the threshold was defined as the lowest level at which waves in the auditory brainstem response could be clearly detected by visual inspection.

### EP measurement

EP recordings were performed in P30 mice under general anaesthesia as described above. The cochlea was exposed using a ventral approach. The bone over the spiral ligament was thinned, and a small opening was created with a pick, allowing access to the endolymphatic compartment (scala media) of the basal turn. The entry of a heat-pulled micropipette electrode filled with 150 mM KCl into the endolymphatic compartment was evidenced by rapid changes in the recorded potentials. The electrode was inserted further until a stable potential was observed. At this point, no alterations were dependent on electrode depth. The signal was amplified through an MEZ-7200 amplifier (Nihonkoden Co., Tokyo, Japan). The direct current potentials were recorded using an A-D converter (USB-6216, National Instruments Japan Co., Tokyo, Japan) coupled to a laptop computer.

### Western blot analysis

Each cochlea was homogenised with a Sonifier S-250A analogue ultrasonic processor (Branson, Danbury, CT, USA). Protein concentrations were measured with a bicinchoninic acid protein assay kit (Thermo Fisher Scientific). The proteins were separated by 12.5% sodium dodecyl sulphate-polyacrylamide gel electrophoresis and were detected with following primary antibodies: anti-Kir4.1 (1:1000; cat. no. APC-035; Alomone Labs). Horseradish peroxidase-conjugated secondary antibodies (Bio-Rad) against primary antibodies, horseradish peroxidase-conjugated anti-β-actin (cat. no. PM053-7; MBL, Nagoya, Japan), and horseradish peroxidase-conjugated anti-GFP (cat. no. PM048-3; MBL) were used at a dilution of 1:5000. The signals were visualised using the ECL System (Bio-Rad). The detected bands were analysed with Image J software (NIH). β-actin was used as internal loading control.

### Laser microdissection

At each stage, whole heads were dissected and stored in RNAlater Stabilization Solution (Thermo Fisher Scientific) at 4°C. The samples were embedded in OCT compound and then sectioned at 10 μm thickness in the plane of the long axis of the cochlear modiolus. Sections were mounted on uncharged slides (Leica, Wetzlar, Germany) and dried at room temperature. The slides were incubated in 95% acetone at −20°C and then dried at room temperature immediately prior to performing LMD. Dissection was performed using the LMD7 system (Leica), according to the method of Pagedar et al. (2006)*.* Samples containing cells were obtained from the cochlear lateral wall. Each slide contained multiple adjacent sections, and we pooled all cells in each category from individual slides onto a single cap.

### Quantitative reverse transcription polymerase chain reaction

Total RNA was extracted from each sample excised with LMD using a micro RNA Extraction kit (Qiagen). After the isolation steps, we quantified RNA using a GeneQuant100 (GE Healthcare Ltd., Amersham, UK), and sample concentrations were equalised. cDNA was synthesised from total RNA with a One-Step PrimeScript RT-PCR Kit (Takara Bio, Otsu, Japan), following the manufacturer's instructions. For qRT-PCR, primers for *Vimentin*, *E-cadherin*, and *TGFβR2* or *GAPDH* (reference gene; Applied Bionics, Foster City, CA, USA) were used. The cDNA was amplified for 40 cycles of denaturation for 15 s at 95°C and annealing for 1 min at 60°C, using a Takara thermal cycler Dice system (model TP960). The relative gene expression was calculated by the standard curve method. Transcript levels for each target were normalised to the expression of *GAPDH*.

### Statistical analysis

The data are presented as the means±standard deviation and two-tailed Student's *t*-tests were performed for statistical comparisons. Differences were considered significant when the *P*<0.05. We used StatView 5.0.1 (SAS Institute Inc.) for all three statistical analyses.
